# Supplementary material for: The limitations of non-mechanistic methods for characterizing pathogen-pathogen interactions: A simulation study
Source: PLoS Comput Biol. 2026 Aug 17;22(8):e1013859. doi: 10.1371/journal.pcbi.1013859 (PMC13480661; doi:10.1371/journal.pcbi.1013859)
Supplement: Supplementary file 2 [file pcbi.1013859.s013.pdf]

First of all, we would like to thank the editor and reviewers for their insightful and constructive comments on the manuscript. We feel that this feedback has greatly improved the manuscript's clarity and accessibility.

Please find below our detailed responses to all individual comments.

Additional Editor Comments:

The reviewers recognize this as a technically competent study addressing an important question—whether standard statistical methods can reliably infer pathogen-pathogen interactions from surveillance data. The manuscript is generally well written, and the simulation framework builds reasonably on prior work. However, the reviewers raise several substantive concerns that need to be addressed before the manuscript can be considered for publication.

1. Insufficient definition of "interaction." Reviewer 1 raises a fundamental conceptual issue: the manuscript conflates different notions of interaction—direct dynamical links between state variables (the type that Granger causality, CCM, and transfer entropy are designed to detect) versus statistical associations between aggregated observed incidence time series. Since the methods are being applied outside their intended domain (inferring links between sums of compartments rather than individual state variables, with many variables unobserved), the negative results for causal inference methods may be expected rather than surprising. The manuscript needs to clearly define and distinguish these interaction concepts throughout, and acknowledge that conclusions about method failure depend heavily on this distinction. This is a major criticism since the premise of this manuscript is exploring limitations across methods.

We are grateful to the reviewers for calling attention to this lack of clarity in the manuscript. We are now much more explicit in the Introduction concerning how we define a pathogen-pathogen interaction throughout this work (lines 64-70). Specifically, the interactions we seek to identify are biological interactions that occur at the level of the individual host. Our goal, then, is to determine whether the outputs of the statistical methods we've tested are reliably indicative of whether or not such interactions are present (lines 133-138).

We agree that the fact that our interaction effect involves several model compartments simultaneously could help explain the difficulty experienced by methods like Granger causality, transfer entropy, and CCM, and that these issues may be even more pronounced due to the use of "observed" data derived from the sums of many compartments. We have revised our Discussion to explicitly acknowledge this, and to contrast the case of pathogen-pathogen interactions with those contexts in which these methods are more successfully applied (e.g., predator-prey dynamics, or other ecological models of population dynamics) (lines 853-867). We have also placed greater emphasis on the fact that, although these methods may not be reliable indicators of pathogen-pathogen interactions, they are still effective in many other fields, and our work is not intended to devalue them (lines 771-774).

Finally, we emphasize the importance of our work up front, despite the fact that some of the assumptions of the methods we have tested are necessarily violated in this context (lines 115-129). In particular, we discuss the preponderance of correlation- and regression-based approaches to study various complex and nonlinear processes in infectious disease epidemiology, despite numerous articles warning against the application of these methods in these contexts. Given that attractive, simple-to-use methods will be applied even when their assumptions are not met (and indeed, some of the methods we've tested are already being applied to study pathogen-pathogen interactions), we feel it is of critical importance to explicitly evaluate their use for questions about pathogen-pathogen interactions.

2. Unobserved compartments and hidden variables. Reviewer 1 also notes that it is unclear how incidence data are constructed from the underlying compartmental model—specifically, how infected and co-infected compartments are aggregated into observed time series. The manuscript does not adequately address the requirement that causal inference methods like Granger causality assume a complete system with no unmeasured confounders. The authors should be transparent about what is observed versus latent, and discuss how aggregation and unobserved variables affect inference.

We have added a subsection to the Methods describing our observation model, which generates the "observed" time series we use for the analyses from the underlying transmission model. In this subsection, we explicitly state that all transmission model states are latent, and that only the observation model's outputs are considered observed (lines 211-227).

The role of unmeasured confounders is an important one, and one that we did not adequately address in the original manuscript. Because this is a simulation study, all extrinsic variables driving transmission and data generation (in our model, only the shared seasonal forcing function) are explicitly modeled and are known. For this reason, we could ensure that this extrinsic confounding was controlled for in our statistical methods where applicable. However, our transmission model describes a nonlinear system with feedback loops. For this reason, latent model states (for example, the proportion susceptible to infection with a given pathogen) will play a prominent role in driving transmission patterns. Because these quantities are rarely observed in real life, we have chosen not to include them as confounders in our simulation study, in order to maintain realism. However, their exclusion could help explain the subpar performance of methods like Granger causality and transfer entropy, which require all confounders to be controlled for. We now explain this in lines 815-820. Furthermore, we discuss additional difficulties with confounding in real-world data in lines 820-824.

3. Potentially unfair comparison across methods (which connects back to point 1). Reviewer 2 notes that the GAM's superior performance may partly reflect its ability to control for pathogen-specific seasonal terms, whereas the other methods account for only a single seasonal confounder. The authors should discuss whether pre-filtering seasonality (e.g., via GAM residuals) before applying causal inference methods would yield a fairer comparison, and provide stronger justification for their modelling choices.

We have assumed the same seasonal forcing function for both pathogens in our model. For this reason, we do not expect a method controlling for seasonal forcing for each pathogen separately (as in GAMs) to have any inherent advantage over Granger causality and transfer entropy, which utilize a single seasonal forcing term.

However, as we discuss in our response to your previous comment, unobserved confounding between cases of pathogens A and B almost certainly exists due to variations in the proportions susceptible over time. It is possible that GAMs, in filtering out patterns in the data, are at least partially accounting for this confounding. Granger causality and transfer entropy, on the other hand, can only control for variables that are explicitly defined by the user. This may give GAMs some degree of advantage over these other methods.

That said, in a nonlinear system, patterns in observed data do not necessarily reflect the patterns in the underlying forcing variables [see Deyle et al. (2016) in PNAS, which is ref. 18 in the revised manuscript]. Therefore, controlling only for the patterns in the observed time series is problematic, and is expected to be less valid and reliable than controlling for the underlying drivers directly. We now discuss this issue in lines 734-736 and 812-818. Ultimately, given these limitations, we were surprised to see that GAMs performed as well as they did.

4. CCM embedding dimension may be too low. The embedding dimension is based on generation intervals, but current cases also depend on susceptible depletion driven by infections much further in the past. A higher embedding dimension based on the duration of immunity may be more appropriate and could affect CCM performance. More broadly, the authors should examine whether other fine-tuning choices across methods may similarly influence the comparison, and provide additional sensitivity analyses where feasible. Where such analyses fall outside the scope of this work, the potential impact of these choices should be explicitly acknowledged in the discussion.

This is a good point, and we have now conducted a sensitivity analysis using higher values for the maximum order of VAR models (Granger causality), history lengths (transfer entropy), and the maximum embedding dimensions and lags (CCM). Specifically, rather than basing these values on generation times, we base them instead on the duration of immunity, in line with your and reviewer 1's point that the number of susceptibles is also an important driver of current cases. This is now explained in detail in the Supplementary Materials (paragraph copied and pasted below for convenience), and these analyses are mentioned throughout the methods (lines 428-429, 481-482, 518-520).

"As described above, we initially chose the upper bounds for several method parameters based on the generation times of influenza and RSV. However, since future cases of a pathogen will depend on both the number of infected and the number of susceptible individuals in the population, and the duration of immunity for our modeled pathogens ranges from 7.2 to 12 months, past cases of a given pathogen may influence cases at time t for significantly longer than the generation time. We therefore repeated our analyses using a value of 37 weeks (the median time to loss of immunity given an exponential distribution with rate = 1/ω, or 1/52.25). Specifically, this value was used as

the history length of the outcome variable (transfer entropy) and the maximum embedding dimension (CCM) for both pathogens; a value of 50 (37 + 13 weeks maximum interaction duration) was used for the maximum order of VAR models (Granger causality), the history length of the predictor variable (transfer entropy), and the maximum lag (CCM).”

We found that these higher values yielded little change in the overall accuracy of the different methods. For CCM only, higher embedding dimensions and lags led to higher sensitivity, but at the cost of roughly equivalent declines in specificity (for the method assessing significance based on convergence in particular, specificity declined to nearly 0). CCM on the effect of pathogen B on A and using seasonal surrogates to infer significance was the exception: sensitivity was increased without a concurrent loss in specificity. However, the resulting specificity was still very low (0.197). For this reason, we maintain our original implementation as the main analysis, but discuss these new results briefly in lines 605-607, as well as in the Supplementary Materials. Results are plotted in Figure S4.

5. Consideration of real-world data. The study relies entirely on simulated data, which is justified based on the purpose of this study. However, as Reviewer 3 points out, incorporating a real-world case study — for instance, using surveillance data for influenza and SARS-CoV-2, which the authors themselves mention as a motivating example — would substantially strengthen the manuscript by grounding the simulation findings in a practical setting and demonstrating whether the identified limitations hold when applied to empirical data. Given the abundance of publicly available surveillance data for respiratory pathogens, including influenza, RSV, and SARS-CoV-2, such an application should be feasible and would considerably enhance the practical relevance of the conclusions. It would also help address questions such as the impact of irregular sampling frequency — which is common in real-world surveillance but not reflected in the regular weekly observations assumed here — on the comparative performance of the methods. If a full analysis is beyond the current scope, the authors should at minimum discuss how their findings would translate to real-world applications where additional complexities (e.g., reporting biases, missing data, irregular sampling frequencies) are present.

We have added an analysis applying each of the tested methods to real-world surveillance data for influenza and RSV from Hong Kong and Canada. We previously used these same data to try to infer the strength and duration of this interaction by fitting a mechanistic transmission model [Kramer et al. (2024) in *Nature Communications*; ref. 3 in the revised manuscript]. Therefore, in addition to comparing results across methods and locations, we were also able to assess whether the methods tested in the current work yielded results consistent with our past modeling work.

As in our simulation study, GAMs showed the best and most consistent performance. Granger causality and transfer entropy were able to identify the effect of influenza on RSV, but could not always find evidence of an effect in the opposite direction. CCM in particular struggled to identify consistent interaction effects across locations. Finally, as we observed with our simulated data, methods did not always agree regarding which

interactions effects were stronger, further emphasizing our conclusion that these methods are limited when it comes to characterizing specific features of interactions.

More detailed methods and results for this analysis can be found in lines 291-311 and 685-701, respectively, and results are visualized in Figure 6. Where relevant, results are also commented on throughout the discussion (e.g., lines 763-769, 797-801, and 839-845).

Reviewers' comments:

Reviewer #1: In this article, the authors investigate the utility of statistical methods for inferring pathoegn-pathogen interactions. In doing so, the authors use mechanistic models to simulate realistic pathogen surveillance data. Then, they compare the following methods for inferring interactions: Pearson correlation, generalized additive model, Granger causality, transfer entropy, and convergent cross-mapping. The authors show that all methods perform poorly, except for the generalized additive model, which has a sensitivity of 85.2% and a specificity of 72.5%. Overall, this work highlights the limitation of statistical approaches for inferring pathogen interactions. It provides a valuable foundation for future studies, particularly given the growing interest in quantifying pathogen interactions across the field. The paper is well written, and the analyses are sound. I have two main concerns.

Thank you for your positive feedback.

First, I was wondering if the comparison between GAM and other methods might be unfair given that GAM allows to control for separate seasonal terms, captured by s_i(WOY), whereas other causal inference methods only account for a single seasonal term, S_t. Reading this paper left me wondering if it would make more sense to first control for the seasonal terms (e.g., through GAM) and then perform causal inference on the residuals. I recognize that this may be beyond the scope of this work, but further elaboration on modeling decisions and references to past work using similar implementations in the context of pathogen interactions would be useful.

We have addressed this point in our response to the editor's third comment above; our response is pasted here for convenience:

"We have assumed the same seasonal forcing function for both pathogens in our model. For this reason, we do not expect a method controlling for seasonal forcing for each pathogen separately (as in GAMs) to have any inherent advantage over Granger causality and transfer entropy, which utilize a single seasonal forcing term.

However, as we discuss in our response to your previous comment, unobserved confounding between cases of pathogens A and B almost certainly exists due to variations in the proportions susceptible over time. It is possible that GAMs, in filtering out patterns in the data, are at least partially accounting for this confounding. Granger causality and transfer entropy, on the other hand, can only control for variables that are explicitly defined by the user. This may give GAMs some degree of advantage over these other methods.

That said, in a nonlinear system, patterns in observed data do not necessarily reflect the patterns in the underlying forcing variables [see Deyle et al. (2016) in PNAS, which is ref. 18 in the revised manuscript]. Therefore, controlling only for the patterns in the observed time series is problematic, and is expected to be less valid and reliable than controlling for the underlying drivers directly. We now discuss this issue in lines 734-736

and 812-818. Ultimately, given these limitations, we were surprised to see that GAMs performed as well as they did."

I was also worried that the the maximum embedding dimension may be too low for the CCM construction. In Supplementary Text, the authors denote ``If generation time for a pathogen is, for example, 3 weeks, this suggests that current case counts can be affected by cases that occurred up to three weeks previously'' and use this argument as a basis for determining the maximum embedding dimension based on the length of generation interval distribution. However, current case counts also depends on the current number of susceptibles, which further depends on infections that occurred many weeks before. Thus, I think it would be more sensible to determine the maximum embedding dimension based on the duration of immunity and therefore consider a higher embedding dimension.

This is an excellent point, and we agree that, at the very least, a sensitivity analysis allowing for higher embedding dimensions is important. Please see our response to the editor's fourth point above (again pasted below for convenience).

"This is a good point, and we have now conducted a sensitivity analysis using higher values for the maximum order of VAR models (Granger causality), history lengths (transfer entropy), and the maximum embedding dimensions and lags (CCM). Specifically, rather than basing these values on generation times, we base them instead on the duration of immunity, in line with yours and reviewer 1's point that the number of susceptibles is also an important driver of current cases. This is now explained in detail in the Supplementary Materials (paragraph copied and pasted below for convenience), and these analyses are mentioned throughout the methods (lines 428-429, 481-482, 518-520).

'As described above, we initially chose the upper bounds for several method parameters based on the generation times of influenza and RSV. However, since future cases of a pathogen will depend on both the number of infected and the number of susceptible individuals in the population, and the duration of immunity for our modeled pathogens ranges from 7.2 to 12 months, past cases of a given pathogen may influence cases at time t for significantly longer than the generation time. We therefore repeated our analyses using a value of 37 weeks (the median time to loss of immunity given an exponential distribution with rate = 1/ω, or 1/52.25). Specifically, this value was used as the history length of the outcome variable (transfer entropy) and the maximum embedding dimension (CCM) for both pathogens; a value of 50 (37 + 13 weeks maximum interaction duration) was used for the maximum order of VAR models (Granger causality), the history length of the predictor variable (transfer entropy), and the maximum lag (CCM).'

We found that these higher values yielded little change in the overall accuracy of the different methods. For CCM only, higher embedding dimensions and lags led to higher sensitivity, but at the cost of roughly equivalent declines in specificity (for the method assessing significance based on convergence in particular, specificity declined to nearly 0). CCM on the effect of pathogen B on A and using seasonal surrogates to infer significance was the exception: sensitivity was increased without a concurrent loss in

specificity. However, the resulting specificity was still very low (0.197). For this reason, we maintain our original implementation as the main analysis, but discuss these new results briefly in lines 605-607, as well as in the Supplementary Materials. Results are plotted in Figure S4."

Minor comments

- Table 1: It would be helpful to add units for the interaction duration

Thank you for catching this. We have updated the Table to specify that these durations are in weeks.

- L186: I was wondering why the authors did not consider longer-term interactions. There have been several

We wanted to focus here on interactions between unrelated pathogens, which we expect to be driven by shorter-lasting mechanisms (e.g., innate immunity). We therefore only tested values for interaction duration that are realistic for this type of interaction. In part, this was to avoid repeating the analyses performed by Cobey and Baskerville (2016) (ref. 36 in the manuscript), who found mixed success for CCM in the specific case of long-lasting cross-immunity. We also note that, in the case of interactions mediated by adaptive immunity, knowledge of the underlying mechanisms provides some degree of prior information concerning the sign and duration of the interaction. This is often not the case for shorter-term interactions, where mechanisms and even the hypothesized sign (i.e., positive or negative) of the interaction may be unknown. Thus, our work serves as a test of various methods for perhaps the most challenging scenarios.

We have added a paragraph to the Methods to outline this decision (lines 236-243).

- L178: It would be helpful to provide more details into how the immunity surges were modeled

More detail on the immunity surges is included in our Supplementary Materials (copied and pasted below); we have added a note to line 207 to explicitly direct interested readers to S1 Text.

"As described in the main text, surges in immunity loss for pathogen A were modeled as occurring once per year, with the exact timing drawn from a normal distribution with mean 12 and standard deviation 4, such that surges occur on average 12 weeks into the outbreak (i.e., during the thirteenth week). Because we are working with purely synthetic data, the exact choice of surge timing is not critical. However, Yaari et al. report that these surges tend to precede the initial rise in epidemic activity each year (1). Thus, it makes sense to place the surges several weeks prior to the peak in transmissibility (here, ϕ = 26), which tends to occur shortly before peak incidence. The size of surges ranged from 5-30% of the recovered population, as described in the main text, and was drawn from a uniform distribution."

- L293: I'm curious why the authors didn't consider using the negative binomial

distribution, instead of a normal distribution. The negative binomial distribution seems like a more natural choice, especially given the negative binomial observation model.

The reviewer raises an important point. For consistency between methods, we log-transformed and centered all data prior to the analysis. After these transformations, the data were no longer discrete, but continuous, thus explaining our choice of a normal, continuous distribution.

- L351: I worry that 20 week order might be too short. In particular, because the 13-week maximum interaction duration represents a mean, the underlying exponential distribution implies that actual interaction times can be substantially longer.

Please see our response above to your second major comment, where we discuss a new sensitivity analysis where we base the maximum order of the VAR models (as well as the history lengths for transfer entropy and the maximum embedding dimensions and lags for CCM) on the duration of immunity for our modeled pathogens, rather than the generation times.

- L379: It would be helpful to provide more mathematical details about how H are calculated. I had trouble understanding the mathematical differences between two H terms.

Getting into the exact mathematics of how both H terms are calculated becomes relatively complicated, and we believe would go far beyond what the average reader will hope to get out of this section. (Note that, despite the similarity in terminology, H here refers to the Shannon entropy, and has nothing to do with the true modeled incidence of each pathogen, which we have also called H.) However, we have added a sentence better explaining what Shannon entropy seeks to measure, which we hope will help readers to have a better conceptual understanding of what is being calculated in Equation 7 (lines 459-463). We have also added an additional explanation of the two H terms in lines 466-468, where we explain what a transfer entropy value above 0 indicates. More mathematical details can be found in, for example, references 84 and 94, both of which are cited in this section.

- L382: this H is missing the main Y_t.

Thank you for noticing this. This is corrected in the updated manuscript (now line 458).

- L386: It is unclear how statistical significance is determined. It would also be helpful to provide an intuitive explanation for why this quantity must be greater than zero. Although the implication of a positive value became clear after several readings and by comparison with Granger causality, many readers would benefit from a more direct and accessible intuition from the authors.

We explain how statistical significance is determined two paragraphs later (lines 486-488, previously lines 404-405). However, to avoid reader confusion, we have removed the word “significantly” from line 466 (previously line 386), and refrain from mentioning significance until we explain how it is determined.

We have added a hopefully more intuitive explanation of the quantity calculated in Equation 7 in lines 466-468.

- L396: I was wondering if the authors had considered using different lengths for the target data. It seems that the results could be sensitive to the choice of target data length.

We considered longer history lengths for transfer entropy in our sensitivity analysis described above, in response to your second major comment.

- L420: It would be helpful to briefly explain Takens' theorem.

We have added a brief definition of Takens' theorem in lines 504-505.

- L483: I'm confused by what this value of 16 represents

Because the reciprocal interaction strength is undefined for an interaction with strength 0, we arbitrarily assigned these interactions the value 16 for this regression analysis. We now explain our reasoning in lines 570-572.

A value of 16 was chosen as it is substantially higher than the next highest value of 4 (for interactions of strength 0.25), reflecting the fact that an interaction with strength 0 (total inhibition) is much stronger than one with strength 0.25 (a 75% reduction in susceptibility). Based on your comment, we repeated the analysis using values of 8 and 64 instead; results were qualitatively similar regardless of the chosen value.

- Figures 3 and 4: It is interesting to see that the sensitivity for most methods are higher for positive interactions (Figure 4). I wondered if Figure 3 would look different if the positive and negative interaction results are plotted separately.

Please see below for the version of Figure 3 where sensitivity for positive (top row) and negative (bottom row) interactions is plotted separately. Note the difference in x-axis limits in the top vs. bottom panels. As in Figure 3, results for the effect of pathogen A on B are shown on the left, and results for the effect of B on A are shown on the right. For both the top and bottom rows, specificity necessarily still refers only to those simulations where no interaction is modeled (i.e., it is defined the same way for the top and bottom panels).

For most methods, sensitivity for positive and negative interactions is similar; the exceptions are correlation coefficients and both implementations of CCM, for which performance is substantially worse for negative interactions.

Since this information is already presented (albeit in a more granular fashion) in Figure 4, we have chosen to keep Figure 3 as-is, in order to convey the overall accuracy of each method.

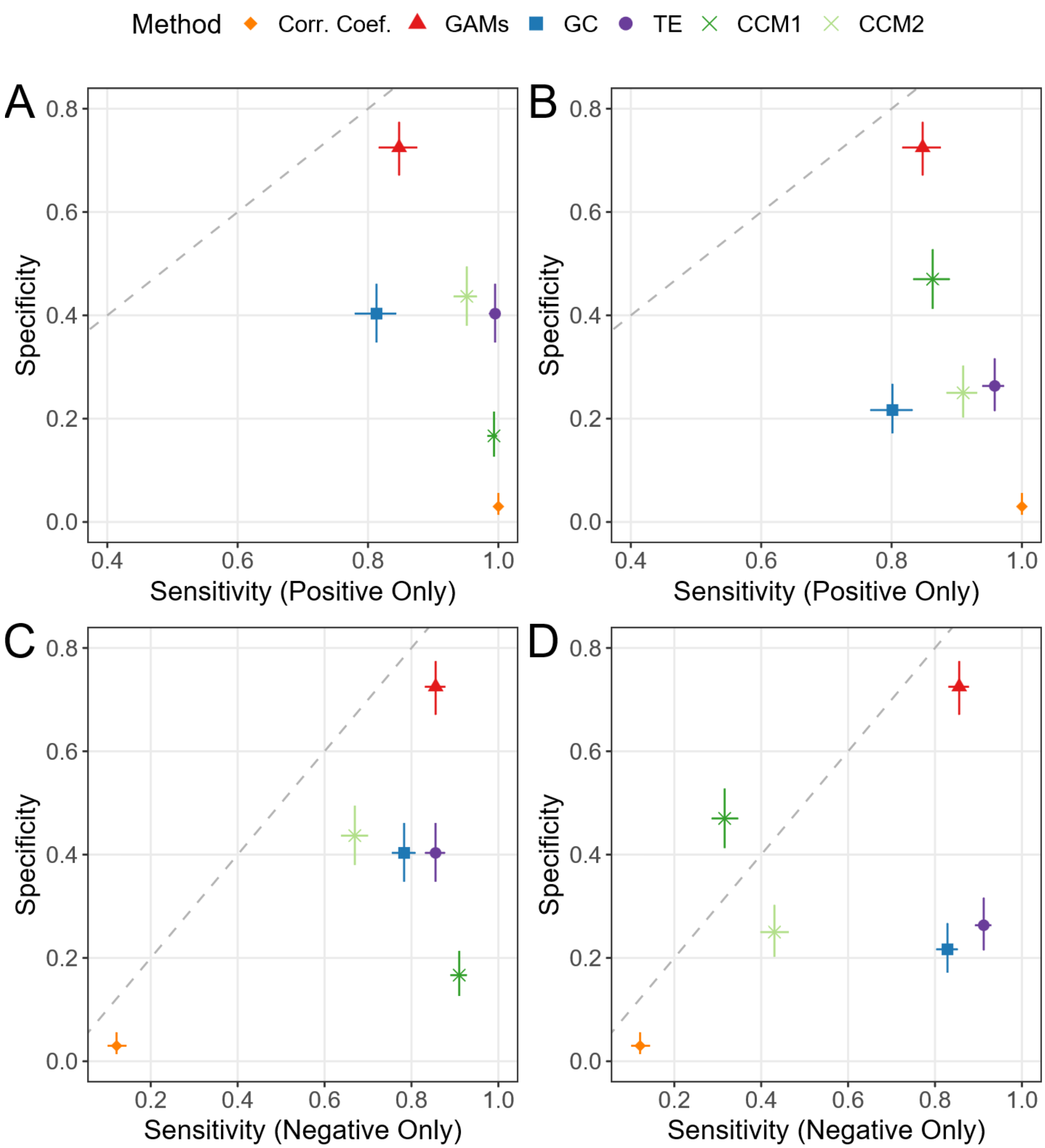

Method
Corr. Coef.
GAMs
GC
TE
CCM1
CCM2
A
B
C
D
Specificity
Sensitivity (Positive Only)
Sensitivity (Negative Only)
0.0
0.2
0.4
0.6
0.8
1.0

Reviewer #2: Inference about potential pathogen–pathogen interactions—where infection with one pathogen either increases or decreases a person's risk of infection or illness due to a second, distinct pathogen—is important for epidemic control. This study shows that four methods frequently used in other fields to identify interactions can yield false positives, incorrectly suggesting interactions when none exist. Through comprehensive simulations, the authors argue that such methods should be rigorously evaluated before being used to draw conclusions about interactions. I agree with the authors that causal inference requires more than the mechanistic assumptions built into these methods to support reliable prediction.

Thank you for your comments.

The authors mention potential interactions between influenza and SARS-CoV-2. Would it be possible to incorporate real-world data of this kind into their simulation study based on synthetic data? I imagine that doing so would greatly increase the value of the current work.

Thank you for your suggestion. We have added an analysis of real-world influenza and RSV data from two countries. For details, please see our response to the editor's comment regarding real-world data above (also pasted here for your convenience).

"We have added an analysis applying each of the tested methods to real-world surveillance data for influenza and RSV from Hong Kong and Canada. We previously used these same data to try to infer the strength and duration of this interaction by fitting mechanistic transmission models [Kramer et al. (2024) in Nature Communications; ref. 3 in the revised manuscript]. Therefore, in addition to comparing results across methods and locations, we were also able to assess whether the methods tested in the current work yielded results consistent with our past modeling work.

As in our simulation study, GAMs showed the best and most consistent performance. Granger causality and transfer entropy were able to identify the effect of influenza on RSV, but could not always find evidence of an effect in the opposite direction. CCM in particular struggled to identify consistent interaction effects across locations. Finally, as we observed with our simulated data, methods did not always agree regarding which interactions effects were stronger, further emphasizing our conclusion that these methods are limited when it comes to characterizing specific features of interactions.

More detailed methods and results for this analysis can be found in lines 291-311 and 685-701, respectively, and results are visualized in Figure 6. Where relevant, results are also commented on throughout the discussion (e.g., lines 763-769, 797-801, and 839-845)."

Reviewer #3:

In this manuscript the authors study pathogen-pathogen interactions as modelled by SEITRSxSEITRS models with two different pathogens and one host, with a seasonal driver. They show that usual time series causal inference methods fail to detect pathogen-pathogen "interactions" but correlative methods can find "interactions". I am putting interactions in quotations marks because there are actually several interaction concepts considered here.

Indeed, from what I can understand pathogen-pathogen "interactions" inferred from observed incidence data do not necessarily map directly to interactions in an ecological theory sense (i.e. state variables directly linked in a dynamical system by a nonzero coefficient) or time series sense (Granger, CCM, transfer entropy), and I believe that this unrecognized distinction between the two concepts may drive a lot of the results found here. I elaborate upon this below. This may also drive why correlative methods find statistical links between pathogen A and B incidences: indirect interactions (X directly affects Y which affects directly Z => X indirectly affects Z) may show up in the data as association. In my opinion this does not mean that methods cannot find interactions, but rather that interaction concepts have been insufficiently defined and delineated.

Thank you for bringing attention to these areas where our manuscript was not specific enough.

We have addressed the original ambiguity in how pathogen-pathogen interactions are defined above in our response to the editor's comments (pasted below for convenience):

"We are now much more explicit in the Introduction concerning how we define a pathogen-pathogen interaction throughout this work (lines 64-70). Specifically, the interactions we seek to identify are biological interactions that occur at the level of the individual host. Our goal, then, is to determine whether the outputs of the statistical methods we've tested are reliably indicative of whether or not such interactions are present (lines 133-138)."

In other words, associations between time series may indicate that an interaction exists, but the association is not itself the interaction. While we acknowledge that the use of the term "interaction" may be somewhat confusing for readers accustomed to reading about "interactions" as the term is used in statistics or in causal inference, we continue to use this term for consistency with the literature on pathogen-pathogen interactions as we define them here.

Your point regarding indirect interactions is an important one: if pathogen A influences the incidence of pathogen B, which then in turn influences the incidence of pathogen C, there is an indirect causal effect of pathogen A on pathogen C, despite a lack of any biological interaction (as defined in the revised Introduction) between these two pathogens. While this cannot explain the results of our simulation study, as we have not included a third, mediating pathogen in the data generation process, it could easily be an issue for real-world data. We now acknowledge the challenge introduced by additional cocirculating pathogens in lines 810-812.

These lack of conceptual and mathematical definitions of interactions aside, much of this work appears to be technically well-done (upon code inspection) and reasonably building upon previous work. But not defining which interactions one is talking about, what are state variables and how they combine into observed variables, is in my opinion seriously misleading readers (albeit likely involuntarily). Time series methods infer *direct* interactions while one tries here to infer indirect or blended ones, if I understood correctly. If the methods are used outside their application domain, this should in my opinion be stated as such very clearly in the abstract, intro and discussion so as not to mislead readers, who want to know for the most part whether statistical methods can be used inside their application domain.

As detailed above in our response to the editor's second point, we have revised the manuscript to clearly define the observation model, and to specify which model quantities are observed and which are latent. We hope that this will help readers to better understand the relationships between model compartments, and how these ultimately lead to the (synthetic) data used for the analyses.

"We have added a subsection to the Methods describing our observation model, which generates the "observed" time series we use for the analyses from the underlying transmission model. In this subsection, we explicitly state that all transmission model states are latent, and that only the observation model's outputs are considered observed (lines 211-227)."

Additionally, we have added equations for the H_1 and H_2 compartments, which track incidence of each pathogen at each time point, to Equation 1 in the Supplementary Materials.

We respond more fully to the rest of the above comment in our reply to your first main point, immediately below.

I now tackle the main points in more detail

1 - What is an interaction? A key aspect of any interaction or causal inference method evaluation is that the interaction (or causal link) concept has to match in the simulation and statistical model for the interaction/causal inference to work. Ref (30), which the authors build upon, define it as a direct dynamical link between compartments in a dynamical system (i.e. there is a link from i to j if a change in state variable X_i modifies dX_j/dt), and show that Granger causality and CCM can find interactions in this context, even though it is more difficult with environmental confounding, including when corrected for (this last point is rightly and well explained by the authors).

Is the pathogen-pathogen interaction that the authors refer to of the same nature, i.e., a non-zero coefficient linking a time derivative of a pathogen-related quantity to another pathogen "density" in a dynamical system? It may be so if the (possibly reconstructed) derivative of the observed variable for pathogen A is a function of the observed variable for pathogen B. Can the authors manipulate the dynamical system to prove that it is actually the case? If not, then the interaction concepts in the dynamical system and

between observed incidences better be given different names. There's no reason to be surprised that Granger and CCM can't find pathogen-pathogen interactions if they have no relationship to the dynamical-system-interactions that Granger analysis and CCM (and transfer entropy) should actually find.

You are correct that the relationship between observed variables from our model is not quite as clear-cut as the direct effect between individual states that methods like Granger causality and CCM are designed to detect. Specifically, rather than a direct effect of one model state on another, the pathogen-pathogen interactions we model represent an effect of both current and past cases of pathogen A on the total subsequent cases of pathogen B (and vice versa). To some extent, this is a similar idea to the one we express in lines 691-703 of the original manuscript (now lines 860-867), although your point is a distinct one. We have addressed this point above in our response to the editor's first comment:

"We agree that the fact that our interaction effect involves several model compartments simultaneously could help explain the difficulty experienced by methods like Granger causality, transfer entropy, and CCM, and that these issues may be even more pronounced due to the use of "observed" data derived from the sums of many compartments. We have revised our Discussion to explicitly acknowledge this, and to contrast the case of pathogen-pathogen interactions with those contexts in which these methods are more successfully applied (e.g., predator-prey dynamics, or other ecological models of population dynamics) (lines 853-867). We have also placed greater emphasis on the fact that, although these methods may not be reliable indicators of pathogen-pathogen interactions, they are still effective in many other fields, and our work is not intended to devalue them (lines 771-774)."

Mathematically, the ODEs for the total incidence rates of pathogen A and B—from which the observed incidence rates are derived through a negative binomial distribution—are as follows (note that the two equations are mirrors of each other, with the indices of the *X* variables swapped):

$$\frac{dH_A}{dt} = \gamma_A(X_{IS} + X_{IE} + X_{II} + X_{IT} + X_{IR})$$
$$\frac{dH_B}{dt} = \gamma_B(X_{SI} + X_{EI} + X_{II} + X_{TI} + X_{RI})$$

The only term common to these two equations is X_{II} (representing the number of co-infected), so that—to the best of our knowledge—there is no easy way of expressing dH_A/dt as a function of H_B, or vice versa. We also note that, in these equations, all the *X* states represent prevalence variables that are typically unobserved. Hence, incidence data represent counts aggregated across multiple, unobserved epidemiological states, reflecting the incomplete observation process characteristic of infectious diseases (at least when only incidence data are available, as we assumed throughout our study).

As you correctly point out, given an interaction involving multiple model compartments, and given an observation model relying on sums of those compartments, the assumptions of Granger causality, transfer entropy, and CCM are not fulfilled. However, we still feel

that testing these methods in the context of pathogen-pathogen interactions is a worthwhile endeavor. First, as Barraquand et al. (ref. 30, which you cite above) point out, these methods are sometimes effective even in contexts where not all assumptions are met (in their case, Granger causality working just as well as CCM when applied to nonlinear systems with an underlying deterministic skeleton). It is possible that the same could be true for interactions involving multiple underlying compartments (indeed, we find that GAMs work decently well, despite there being good theoretical reason to expect regression-based methods to fail (lines 732-736)). Second, these methods (as well as other, simpler statistical methods whose assumptions are more heavily violated) are already used in the interactions literature (see, for example, refs. 38 and 39 in the revised manuscript). For this reason, formal testing of these methods is urgently needed. We now make this second point in the revised Introduction (lines 115-129). Please see also our response to a similar comment by the editor:

"Finally, we emphasize the importance of our work up front, despite the fact that some of the assumptions of the methods we have tested are necessarily violated in this context (lines 115-129). In particular, we discuss the preponderance of correlation- and regression-based approaches to study various complex and nonlinear processes in infectious disease epidemiology, despite numerous articles warning against the application of these methods in these contexts. Given that attractive, simple to use methods will be applied even when their assumptions are not met (and indeed, some of the methods we've tested are already being applied to study pathogen-pathogen interactions), we feel it is of critical importance to explicitly evaluate their use for questions about pathogen-pathogen interactions."

2 - The authors have actually not clarified which time series compartments are observed, i.e. what constitutes incidence data, and how this choice may affect the "interaction" inference. From Fig. 1 and 2 I tend to deduce that they have summed up all infected stages with pathogen A and all infected stages with pathogen B (it was not clear from the code). But how to handle coinfection? Is X_II counted in each pathogen? Or is it removed in each case? How does that choice to include or remove coinfections affects statistical inference?

I want to stress again that there is zero theoretical guarantee that an interaction inference method (like Granger or CCM) designed to infer all links between compartments in the full dynamical system (if it is observed) will actually infer links between sums of compartments, while other compartments stay unobserved. In fact, this recent review [Shojaie, A., & Fox, E. B. (2022). Granger causality: A review and recent advances. Annual Review of Statistics and Its Application, 9(1), 289-319.] mentions that Granger analysis should be applied when there is a "Complete system: All relevant variables are assumed to be observed and included in the analysis—i.e., there are no unmeasured confounders." These challenges may be vaguely alluded to in the present manuscript l. 698-703 but I believe that the authors should be much more precise regarding what variables they observe, how they might combine state variables, and which state variables are unobserved.

As described in more detail above, we have added a subsection to the Methods to describe the observation model, which produces the synthetic "data" on which our

analyses are run (lines 211-227). Individuals in the *X_II* compartment are indeed counted as cases of both pathogens; this is done to mimic real-world outbreak data, which typically report the number of cases of each pathogen, but often do not report how many of these infections were mono- vs. coinfections.

Regarding the challenge of unobserved confounders, please see our response to the editor's second point (pasted for your convenience here):

"The role of unmeasured confounders is an important one, and one that we did not adequately address in the original manuscript. Because this is a simulation study, all extrinsic variables driving transmission and data generation (in our model, only the shared seasonal forcing function) are explicitly modeled and are known. For this reason, we could ensure that this extrinsic confounding was controlled for in our statistical methods where applicable. However, our transmission model describes a nonlinear system with feedback loops. For this reason, latent model states (for example, the proportion susceptible to infection with a given pathogen) will play a prominent role in driving transmission patterns. Because these quantities are rarely observed in real life, we have chosen not to include them as confounders in our simulation study, in order to maintain realism. However, their exclusion could help explain the subpar performance of methods like Granger causality and transfer entropy, which require all confounders to be controlled for. We now explain this in lines 815-820. Furthermore, we discuss additional difficulties with confounding in real-world data in lines 820-824."

3 - Interactions between original pathogen state variables may or may not emerge as correlations between observed variables at new timelags as they blend multiple processes. This makes finding that correlative methods (using GAMs, whose equations might also be presented more precisely, with better indexing) infer "statistical links" between pathogen A and B time series better than interaction inference methods less surprising, I think. I am not saying this is what happens here---but it is possible.

The fact that pathogen-pathogen interactions do not necessarily lead to intuitive correlations between observed outbreak data is true, and we make this point ourselves in lines 87-95 (originally lines 81-88). In fact, this point strongly motivates our study: if correlative approaches are unreliable, perhaps more complex methods, specifically developed to infer causality, could be more successful. It is our understanding that CCM in particular is skilled at identifying underlying relationships, even when the underlying (nonlinear) processes do not produce correlations between observed variables. (Although, as you rightly point out and as we now acknowledge, the assumptions of methods like CCM are not fully fulfilled in the case of pathogen-pathogen interactions.) Ultimately, we were surprised to find that GAMs performed so well here, given that complex interaction processes do not guarantee the existence of a correlation between observed case counts (lines 729-733).

Note that direct interactions cannot always be recovered if only part of the system is observed. This connects to the Granger causality review paper mentioned above, but also ecological theory such as Schaffer linking a "true" ecosystem to a more simplified representation with missing variables
https://esajournals.onlinelibrary.wiley.com/doi/10.2307/2937321 Some literature on

ecological time series models shows which coefficients can be estimated when only part of the system is observed [e.g. Abbott et al. 2009 https://esajournals.onlinelibrary.wiley.com/doi/full/10.1890/08-0487.1]. The statistical literature on latent variable models in statistics probably makes similar points.

We have partially addressed this point above under your second main point, where we discuss unobserved confounding. Furthermore, potential issues with analyses applied to observed data, whose use will naturally exacerbate any existing violations of a method's assumptions, are now addressed throughout lines 797-801 and 808-812.

Beyond this, it is possible that the methods we've tested would indeed perform better if applied to counts obtained from the unobserved states of the transmission model. However, we have not tested this using simulated values, simply because it is not feasible in the real world, and the main goal of our work was to identify promising methods for analyzing pathogen-pathogen interactions on the basis of real-world data (lines 140-141). That said, we now more explicitly justify our decision to use imperfect data in our analyses (see lines 115-129, 212-219, and 806-808).

Perhaps some algebra can be attempted in the present paper so as to guess which type of relationship could be expected between pathogen incidences, and how the direct interactions in the dynamical system might combine with other parameters in the compound interaction parameters inferred from observed variables, with the various methods considered. One useful starting point may be that the derivative of the sum of infected compartments with pathogen A is the sum of derivatives for individual compartements related to pathogen A, for which expressions are presented in the SI. Another idea would be to approximate the continuous-time dynamical systems (linearized if need be) by a VAR(1) model for the full state variables, leading plausibly to a different time series representation of the two pathogen incidences.

Please see our response to your first main point above, where we have attempted to manipulate the model equations to more clearly show how the interaction effect impacts observed cases of each pathogen.

Regarding analyses involving the unobserved compartments from the transmission model, we reiterate that, while such analyses may prove more accurate than analyses involving observed time series, they are unfortunately not possible in real life. Therefore, we have limited our work to only those analyses that can plausibly be performed on real-world data.
