# Supplementary material for: The limitations of non-mechanistic methods for characterizing pathogen-pathogen interactions: A simulation study
Source: PLoS Comput Biol. 2026 Aug 17;22(8):e1013859. doi: 10.1371/journal.pcbi.1013859 (PMC13480661; doi:10.1371/journal.pcbi.1013859)
Supplement: Supplementary file 3 [file pcbi.1013859.s014.pdf]

We appreciate the additional feedback on our revised manuscript. We hope that the clarifications and discussions we've added to the text will help to dispel any remaining ambiguity. Please find below our detailed responses to all comments.

Reviewers' comments:

Reviewer #3: This a second review. The authors have made a number of modifications to the manuscript in response to my previous comments, with some improvements. However, I believe that the manuscript still falls a bit short regarding the need for precise vocabulary and mathematical equations, and I encourage the authors to try to be more precise whenever possible. What I expected was a mathematical definition of "interaction" in a pathogen-pathogen sense (using the ODE model), with comparison to the sort of interaction concept used in the methods, including mathematical or computational developments to better compare the notions explored here, with changes to the wording to avoid ambiguities. The authors added caveats and a bit of methodology details but there was no major overhaul, in my opinion.

Please see our responses to your more detailed comments below, which we hope will make the mathematical details of the modeled pathogen-pathogen interaction clearer.

Regarding a comparison to the interaction concepts used by the statistical methods, respectfully, we struggle to see how a direct comparison is possible. The non-mechanistic methods we've tested look for certain relationships between time series data, and from those relationships, infer the existence (or absence) of causal mechanisms. For example, Granger causality and transfer entropy both make the underlying assumption that predictability between time series implies the existence of a causal mechanism (given, of course, that some additional assumptions are met). (Notably, as we point out in lines 434-43 (previously lines 420-422), this underlying assumption is not necessarily valid.) In other words, the mathematics employed do not directly describe the nature of the interaction effect being studied.

This is in stark contrast to a mechanistic transmission model like the one we use in this work, which mathematically describes a very specific, mechanistic interaction effect between two pathogens (again, see below for more discussion of the exact nature of this interaction). Any relationship between the time series of observed incidence for the two pathogens emerges naturally from the underlying mechanistic model, and, as we emphasize throughout the manuscript (see, for example, lines 95-99, 360-363, and 746-747 in the revised version), nonlinearity and confounding make it difficult to predict what this relationship will look like. Therefore, although we can assess whether the resulting patterns in the pathogen incidence data allow for the underlying interaction to be identified using a range of methods (which is our goal throughout this manuscript), we can't make any statements regarding the comparability of the interaction concepts in our model vs. the various methods applied. (We can, of course, discuss whether the tested methods are applicable to the type of incidence data we are confronting them with; this we have discussed at length in our previous responses.)

With all that said, we hope that some of our responses to your more detailed feedback below will assuage your concerns about the comparability between interactions as defined by our model and by the statistical methods used for the analyses.

I still do not know which parameters encapsulate the biological interaction between strains (this is not written anywhere as far as I can see, and the reader should not have to dig into the SI to see this).

The strength of the biological interaction (i.e., the extent to which infection with one pathogen reduces or amplifies the chances of infection with the other pathogen upon exposure) is controlled by $\theta_{\lambda 1}$ (for the impact of pathogen 1 on pathogen 2) and by $\theta_{\lambda 2}$ (for the impact of pathogen 2 on pathogen 1). When these parameters are 1.0, no interaction occurs. Positive interactions (i.e., where infection with one pathogen increases susceptibility to the other) are modeled with values above 1.0, and negative interactions (i.e., where infection with one pathogen decreases susceptibility to the other) are modeled with values between 0 and 1.0 (where 0 indicates total inhibition of the second pathogen encountered). In epidemiological terms, the parameter $\theta_{\lambda 1}$ thus represents a relative risk, with values above 1 indicating an increased risk (with, e.g., a value of 1.1 meaning that active or recent infection with virus 1 increases the risk of infection with virus 2 by 10%), while a value below 1 indicates a decreased risk.
The interaction strength parameter $\theta_{\lambda 1}$ specifically modulates the transitions between X_IS and X_IE and between X_TS and X_TE, while the parameter $\theta_{\lambda 2}$ modulates the transitions between X_SI and X_EI and between X_ST and X_ET (see Figure 1).

The extent to which this effect persists after recovery is modeled by δ_1 (for the impact of pathogen 1 on pathogen 2) and δ_2 (for the impact of pathogen 2 on pathogen 1); specifically, these parameters represent the inverse of the duration of the transient post-infectious period T, during which individuals are no longer infected with the initial virus encountered but still subject to the effects of the interaction. The interaction duration paramaters δ_1 and δ_2 modulate the rate of transition from X_TS to X_RS and from X_ST to S_SR, respectively (Figure 1).

This information, including the exact roles of $\theta_{\lambda 1}$, $\theta_{\lambda 2}$, δ_1, and δ_2 in producing a pathogen-pathogen interaction in our model, is detailed in lines 160-175 (previously lines 154-163), and schematically represented in Figure 1.

We have attempted to clarify the nature of the interaction, as well as how these parameters are used to model it, by explicitly stating which transitions between states in the underlying transmission model are controlled by each interaction parameter (lines 168-170 and 172-173). We also now explicitly reference the interaction parameters in the legend to Figure 1, and bold parameters involved in the interaction in Table 1.

The definitions introduced to clarify the concepts in the introduction stay rather vague. I try to explain below why I still believe this to be problematic and what can be done to be more precise.

Please see below for our responses to each of your individual points.

Mathematical interaction concepts

l. 67-68 the authors write now to clarify what interaction concepts they are using "In this work, we use the term to refer exclusively to interactions arising due to biological, within-host mechanisms." as opposed to statistical correlations (presumably using Pearson, though this is implicit) which many quantitative biologist now refer to as "statistical associations". There are still a very large number of ways that "interaction" in this sentence can be interpreted. I meant instead something near-mathematical like "when state variable X from the model is increased, state variable Y's derivative dY/dt is decreased" or alternatively, which parameters of the ODE model (which hopefully represents decently well real-life dynamic processes) encapsulate the biological interaction.

You are correct that, at this point in the manuscript, the exact definition of a pathogen-pathogen interaction remains broad. This was intentional: because pathogen-pathogen interactions in real life occur between a wide range of pathogens, the mechanisms involved in these interactions and the influence they have on transmission dynamics are also highly varied, and it is therefore impossible to provide a one-size-fits-all, mathematical description of how all pathogen-pathogen interactions operate. At this point in the introduction, where we are still introducing the general concept of a pathogen-pathogen interaction to the reader, we prefer to keep our definitions general, in order to encompass this diversity. However, we are now more explicit about the fact that, even limiting ourselves to “interactions arising due to biological, within-host mechanisms,” our definition remains quite general, and applies to a wide variety of more specific interaction effects (lines 68-69). We have also added a sentence to explain that the exact effect of an interaction (i.e., whether it influences transmission, severity, or both, which will influence how the interaction is modeled mathematically) can vary depending on the underlying pathogens and mechanisms (lines 71-74). Furthermore, we have been more specific about the nature of the interaction effect we test here in the final paragraph of the Introduction (lines 142-144).

That said, we have updated the Methods to be more explicit about the nature of the interaction. In particular, as noted in our response to your previous point, we describe how specific transition rates between state variables in our transmission model are driven by the interaction parameters (lines 168-170 and 172-173).

The authors introduced in the response letter variables H_A and H_B, which first sum and then integrate between t-1 and t state variables X_IS, X_IE, X_II, X_IT, X_IR before being observed with error. They also write this now in the observation model l. 233. Such presentation is better than the previous version but still unsatisfactory: we have a precise formulation of the state variables that are available for statistical inference (the interaction concept that the time series methods might infer) but no clear idea of what constitute a biological interaction in the mechanistic model, and importantly, what is the link to be expected between the two.

Interesting methodological avenues to better understand and compare the interaction concepts here would be:

- what parameters of the ODE system generate the interaction between strains, i.e. what

parameters do we have to set to zero so that the interaction between pathogens disappear?
And, to better understand how the information gets degraded as one moves from the "mechanistic" (compartmental ODE) representation to the statistical time series representation (without latent variables), one could explore:
- what is the relationship between the integrated H_A(t) and the "instantaneous" sum (X_IS + X_IE + X_II + X_IT + X_IR)? (e.g. plot a graph of these two quantities using the ODE system)
- what are the state variables that contribute most to the sum, if any?

As explained above, the parameters involved in the modeled interaction and their influence on the underlying transmission dynamics are detailed in lines 160-175 (previously lines 154-163). As stated in lines 167-168 (previously lines 161-162), setting both θ_λ terms to 1.0 results in a model with no pathogen-pathogen interaction.

Your suggestions for gaining more insight into the relationship beween H1 (or H2) and the sum of the underlying state variables used to generate H1 (X_IS + X_IE + X_II + X_IT + X_IR) are good ones, and the corresponding plots can now be found as Figure S9.

We find that there is a roughly linear relationship between infection incidence (H1) and prevalence (X_IS + X_IE + X_II + X_IT + X_IR) (Figure S9A), although the extent to which they differ varies seasonally (presumably due to the delay between infection and reporting). These patterns are consistent across interaction strengths and durations. Overall, these plots suggest that there is not much degradation of information caused by converting from prevalence to incidence (although we note that the “observed” incidence used for all analyses is the total incidence multiplied by a reporting rate, so there is still substantial underreporting).

As your final suggestion above recognizes, not all state variables contribute directly to the interaction (see Figure 1 and lines 160-162 and 170-172). While the observed number of cases of pathogen 1 (H1) will necessarily be derived from a sum of all five model compartments containing individuals infected with pathogen 1, only those in X_IS can be influenced by the interaction effect, as individuals in the other four compartments (X_IE, X_II, X_IT, and X_IR) are or have already been infected with pathogen 2. On a similar note, individuals in X_TS are affected by the interaction, but, since they are no longer infected with pathogen 1, they will not be represented by H1 at time *t*, but rather by past values of H1. For this reason, we have plotted out not only the contribution of X_IS (X_SI) to X_IS + X_IE + X_II + X_IT + X_IR (X_SI + X_EI + X_II + X_TI + X_RI) over time, but also the magnitude of X_TS (X_ST) over time.

We find that 1) individuals in X_IS make up about 40% of the total individuals infected with pathogen 1 at a given time point, on average, with seasonal variation, and 2) X_TS ranges from being considerably smaller than H1 for shorter-duration interactions to being sometimes several times higher than H1 for longer-duration interactions.

That said, we are not convinced that either of these observations represents an inherent problem for method performance. First of all, although four of the five compartments

included in the total prevalence do not contribute directly to the interaction, they all work together to define the force of infection, which will have downstream effects on X_IS, and therefore on the contribution of the interaction to the overall outbreak dynamics. Second, while those in X_TS at time *t* are not included in H1 at time *t*, they are included in H1 at previous timesteps, since they represent past infections. Since the statistical methods we've tested typically incorporate some sort of lag, these individuals are still considered in the analysis. Finally, the size of the X_TS compartment relative to H1 is largest for longer-lasting interactions. If this were a problem for the methods, we would expect performance to suffer in these scenarios. However, we consistently see the opposite: methods performed best when interaction effects were stronger and longer-lasting.

All of these points are discussed in lines 846-866 of the revised manuscript.

Additional wording suggestions

Abstract "simple, purely statistical methods" -> "basic correlative methods" [not sure a "purely statistical" method exists] or perhaps "standard correlations". Same remark for l. 66-67 of the introduction.

Thank you for the suggestion. We agree that it is important to be clear about the types of methods and relationships we are talking about. We have changed "purely statistical" to "correlative" in the abstract (line 24), and "purely statistical associations" to "purely correlative relationships" in the Introduction (lines 66-67).

Other suggestions

Granger causality is a general concept linked to the increase in variance explained / decrease in residual variance when including a given state variable as covariate in a time series model for another state variable's future values. It could in theory be applied to models including latent variables written in POMP or something else, i.e., there could be other ways to assess Granger causality than with the VAR-based representation used here, although the VAR(p) representation is arguably the most common flavour of such models (and is indeed not appropriate for latent variables). Granger causality as a concept (picking causal state variable based on the fact that they decrease residual variance in state variables to be explained) is very much compatible with mechanistic or latent variable (state-space) modelling. This could be mentioned somewhere. See also your ref 90 Barnett, L., & Seth, A. K. (2015). Granger causality for state-space models. Physical Review E, 91(4), 040101. [This may appear to run counter to the previous statement on confounders by Shojaie & Fox but the latter referred to classical VAR-based Granger Analysis, not latent variable modelling]

Thank you for pointing this out. We now mention the existence of formulations of Granger causality specifically designed for state-space modeling in line 421-422. (However, we note here that the method discussed by Barnett & Seth appears to apply specifically to linear models, whereas our transmission model is nonlinear.)

The GAM equation l. 386 does not make very clear how time enters the equation. Perhaps making clear the dimensions of the matrices may help.

In the fitted GAM, time only enters the equation in the form of the smooth on week of the year. In other words, μ at a given time t is defined according to only the intercept (which is not time-dependent) and the value of the smooth for that week (where week is between 1 and 53) – the week-of-the-year-specific values of the smooth do not vary from year to year. This is done so that we can remove any regular seasonal cycle from the data, leaving only the residual variability in both time series over time.

We have now clarified this in the text (lines 381-383). Additionally, we have added subscripts to the relevant equations in lines 388-391, as well as an explicit reference to the basis dimension of the smooth on the week of the year, to better indicate how time comes into play. The updated model equations are pasted below for your convenience. This hopefully also makes the dimensions of the matrices more obvious: these are dependent on μ, which is the length of the time series of pathogen case counts.

$$\boldsymbol{X_t} \sim N_2(\boldsymbol{\mu_t}, \boldsymbol{\Sigma})$$

$$\boldsymbol{\mu_t} = [\mu_{1,t}, \mu_{2,t}]^T$$

$$\boldsymbol{\Sigma} = \begin{bmatrix} \sigma_1^2 & \rho\sigma_1\sigma_2 \\ \rho\sigma_1\sigma_2 & \sigma_2^2 \end{bmatrix}$$

$$\mu_{i,t} = \alpha_i + s_i(WOY_t, k = 53) \quad (3)$$
