# Supplementary material for: The limitations of non-mechanistic methods for characterizing pathogen-pathogen interactions: A simulation study
Source: PLoS Comput Biol. 2026 Aug 17;22(8):e1013859. doi: 10.1371/journal.pcbi.1013859 (PMC13480661; doi:10.1371/journal.pcbi.1013859)
Supplement: Peer review history

# Peer review: Decision Letter 0

**DOI:** 10.1371/journal.pcbi.1013859.r001

**Authors:** Roger Kouyos, Burcu Tepekule

17 Feb 2026

Limitations of non-mechanistic methods for characterizing pathogen-pathogen interactions: A simulation study

PLOS Computational Biology

Dear Dr. Kramer,

Thank you for submitting your manuscript to PLOS Computational Biology. After careful consideration, we feel that it has merit but does not fully meet PLOS Computational Biology's publication criteria as it currently stands. Therefore, we invite you to submit a revised version of the manuscript that addresses the points raised during the review process.

Please submit your revised manuscript by 19.04.2026. If you will need more time than this to complete your revisions, please reply to this message or contact the journal office at ploscompbiol@plos.org. When you're ready to submit your revision, log on to https://www.editorialmanager.com/pcompbiol/ and select the 'Submissions Needing Revision' folder to locate your manuscript file.

Please include the following items when submitting your revised manuscript:

* A letter that responds to each point raised by the editor and reviewer(s). You should upload this letter as a separate file labeled 'Response to Reviewers'. This file does not need to include responses to formatting updates and technical items listed in the 'Journal Requirements' section below.

* A marked-up copy of your manuscript that highlights changes made to the original version. You should upload this as a separate file labeled 'Revised Manuscript with Track Changes'.

* An unmarked version of your revised paper without tracked changes. You should upload this as a separate file labeled 'Manuscript'.

If you would like to make changes to your financial disclosure, competing interests statement, or data availability statement, please make these updates within the submission form at the time of resubmission. Guidelines for resubmitting your figure files are available below the reviewer comments at the end of this letter

We look forward to receiving your revised manuscript.

Kind regards,

Burcu Tepekule

Guest Editor

PLOS Computational Biology

Roger Kouyos

Section Editor

PLOS Computational Biology

**Additional Editor Comments:**

The reviewers recognize this as a technically competent study addressing an important question—whether standard statistical methods can reliably infer pathogen-pathogen interactions from surveillance data. The manuscript is generally well written, and the simulation framework builds reasonably on prior work. However, the reviewers raise several substantive concerns that need to be addressed before the manuscript can be considered for publication.

1. Insufficient definition of "interaction." Reviewer 1 raises a fundamental conceptual issue: the manuscript conflates different notions of interaction—direct dynamical links between state variables (the type that Granger causality, CCM, and transfer entropy are designed to detect) versus statistical associations between aggregated observed incidence time series. Since the methods are being applied outside their intended domain (inferring links between sums of compartments rather than individual state variables, with many variables unobserved), the negative results for causal inference methods may be expected rather than surprising. The manuscript needs to clearly define and distinguish these interaction concepts throughout, and acknowledge that conclusions about method failure depend heavily on this distinction. This is a major criticism since the premise of this manuscript is exploring limitations across methods.

2. Unobserved compartments and hidden variables. Reviewer 1 also notes that it is unclear how incidence data are constructed from the underlying compartmental model—specifically, how infected and co-infected compartments are aggregated into observed time series. The manuscript does not adequately address the requirement that causal inference methods like Granger causality assume a complete system with no unmeasured confounders. The authors should be transparent about what is observed versus latent, and discuss how aggregation and unobserved variables affect inference.

3. Potentially unfair comparison across methods (which connects back to point 1). Reviewer 2 notes that the GAM's superior performance may partly reflect its ability to control for pathogen-specific seasonal terms, whereas the other methods account for only a single seasonal confounder. The authors should discuss whether pre-filtering seasonality (e.g., via GAM residuals) before applying causal inference methods would yield a fairer comparison, and provide stronger justification for their modelling choices.

4. CCM embedding dimension may be too low. The embedding dimension is based on generation intervals, but current cases also depend on susceptible depletion driven by infections much further in the past. A higher embedding dimension based on the duration of immunity may be more appropriate and could affect CCM performance. More broadly, the authors should examine whether other fine-tuning choices across methods may similarly influence the comparison, and provide additional sensitivity analyses where feasible. Where such analyses fall outside the scope of this work, the potential impact of these choices should be explicitly acknowledged in the discussion.

5. Consideration of real-world data. The study relies entirely on simulated data, which is justified based on the purpose of this study. However, as Reviewer 3 points out, incorporating a real-world case study — for instance, using surveillance data for influenza and SARS-CoV-2, which the authors themselves mention as a motivating example — would substantially strengthen the manuscript by grounding the simulation findings in a practical setting and demonstrating whether the identified limitations hold when applied to empirical data. Given the abundance of publicly available surveillance data for respiratory pathogens, including influenza, RSV, and SARS-CoV-2, such an application should be feasible and would considerably enhance the practical relevance of the conclusions. It would also help address questions such as the impact of irregular sampling frequency — which is common in real-world surveillance but not reflected in the regular weekly observations assumed here — on the comparative performance of the methods. If a full analysis is beyond the current scope, the authors should at minimum discuss how their findings would translate to real-world applications where additional complexities (e.g., reporting biases, missing data, irregular sampling frequencies) are present.

**Journal Requirements:**

If the reviewer comments include a recommendation to cite specific previously published works, please review and evaluate these publications to determine whether they are relevant and should be cited. There is no requirement to cite these works unless the editor has indicated otherwise.

1) We ask that a manuscript source file is provided at Revision. Please upload your manuscript file as a .doc, .docx, .rtf or .tex. If you are providing a .tex file, please upload it under the item type u2018LaTeX Source Fileu2019 and leave your .pdf version as the item type u2018Manuscriptu2019.

2) Please provide an Author Summary. This should appear in your manuscript between the Abstract (if applicable) and the Introduction, and should be 150-200 words long. The aim should be to make your findings accessible to a wide audience that includes both scientists and non-scientists. Sample summaries can be found on our website under Submission Guidelines:

https://journals.plos.org/ploscompbiol/s/submission-guidelines#loc-parts-of-a-submission

3) Please upload all main figures as separate Figure files in .tif or .eps format. For more information about how to convert and format your figure files please see our guidelines:

https://journals.plos.org/ploscompbiol/s/figures

4) Please send a completed 'Competing Interests' statement, including any COIs declared by your co-authors. If you have no competing interests to declare, please state "The authors have declared that no competing interests exist". Otherwise please declare all competing interests beginning with the statement "I have read the journal's policy and the authors of this manuscript have the following competing interests"

**Reviewers' comments:**

Reviewer's Responses to Questions

**Comments to the Authors:**

**Please note here if the review is uploaded as an attachment.**

Reviewer #1: In this article, the authors investigate the utility of statistical methods for inferring pathoegn-pathogen interactions. In doing so, the authors use mechanistic models to simulate realistic pathogen surveillance data. Then, they compare the following methods for inferring interactions: Pearson correlation, generalized additive model, Granger causality, transfer entropy, and convergent cross-mapping. The authors show that all methods perform poorly, except for the generalized additive model, which has a sensitivity of 85.2% and a specificity of 72.5%. Overall, this work highlights the limitation of statistical approaches for inferring pathogen interactions. It provides a valuable foundation for future studies, particularly given the growing interest in quantifying pathogen interactions across the field. The paper is well written, and the analyses are sound. I have two main concerns.

First, I was wondering if the comparison between GAM and other methods might be unfair given that GAM allows to control for separate seasonal terms, captured by s_i(WOY), whereas other causal inference methods only account for a single seasonal term, S_t. Reading this paper left me wondering if it would make more sense to first control for the seasonal terms (e.g., through GAM) and then perform causal inference on the residuals. I recognize that this may be beyond the scope of this work, but further elaboration on modeling decisions and references to past work using similar implementations in the context of pathogen interactions would be useful.

I was also worried that the the maximum embedding dimension may be too low for the CCM construction. In Supplementary Text, the authors denote ``If generation time for a pathogen is, for example, 3 weeks, this suggests that current case counts can be affected by cases that occurred up to three weeks previously'' and use this argument as a basis for determining the maximum embedding dimension based on the length of generation interval distribution. However, current case counts also depends on the current number of susceptibles, which further depends on infections that occurred many weeks before. Thus, I think it would be more sensible to determine the maximum embedding dimension based on the duration of immunity and therefore consider a higher embedding dimension.

Minor comments

- Table 1: It would be helpful to add units for the interaction duration

- L186: I was wondering why the authors did not consider longer-term interactions. There have been several

- L178: It would be helpful to provide more details into how the immunity surges were modeled

- L293: I'm curious why the authors didn't consider using the negative binomial distribution, instead of a normal distribution. The negative binomial distribution seems like a more natural choice, especially given the negative binomial observation model.

- L351: I worry that 20 week order might be too short. In particular, because the 13-week maximum interaction duration represents a mean, the underlying exponential distribution implies that actual interaction times can be substantially longer.

- L379: It would be helpful to provide more mathematical details about how H are calculated. I had trouble understanding the mathematical differences between two H terms.

- L382: this H is missing the main Y_t.

- L386: It is unclear how statistical significance is determined. It would also be helpful to provide an intuitive explanation for why this quantity must be greater than zero. Although the implication of a positive value became clear after several readings and by comparison with Granger causality, many readers would benefit from a more direct and accessible intuition from the authors.

- L396: I was wondering if the authors had considered using different lengths for the target data. It seems that the results could be sensitive to the choice of target data length.

- L420: It would be helpful to briefly explain Takens' theorem.

- L483: I'm confused by what this value of 16 represents

- Figures 3 and 4: It is interesting to see that the sensitivity for most methods are higher for positive interactions (Figure 4). I wondered if Figure 3 would look different if the positive and negative interaction results are plotted separately.

Reviewer #2: Inference about potential pathogen–pathogen interactions—where infection with one pathogen either increases or decreases a person’s risk of infection or illness due to a second, distinct pathogen—is important for epidemic control. This study shows that four methods frequently used in other fields to identify interactions can yield false positives, incorrectly suggesting interactions when none exist. Through comprehensive simulations, the authors argue that such methods should be rigorously evaluated before being used to draw conclusions about interactions. I agree with the authors that causal inference requires more than the mechanistic assumptions built into these methods to support reliable prediction.

The authors mention potential interactions between influenza and SARS-CoV-2. Would it be possible to incorporate real-world data of this kind into their simulation study based on synthetic data? I imagine that doing so would greatly increase the value of the current work.

Reviewer #3: Review of Limitations of non-mechanistic methods for characterizing pathogen-pathogen interactions: A simulation study

In this manuscript the authors study pathogen-pathogen interactions as modelled by SEITRSxSEITRS models with two different pathogens and one host, with a seasonal driver. They show that usual time series causal inference methods fail to detect pathogen-pathogen "interactions" but correlative methods can find "interactions". I am putting interactions in quotations marks because there are actually several interaction concepts considered here.

Indeed, from what I can understand pathogen-pathogen "interactions" inferred from observed incidence data do not necessarily map directly to interactions in an ecological theory sense (i.e. state variables directly linked in a dynamical system by a nonzero coefficient) or time series sense (Granger, CCM, transfer entropy), and I believe that this unrecognized distinction between the two concepts may drive a lot of the results found here. I elaborate upon this below. This may also drive why correlative methods find statistical links between pathogen A and B incidences: indirect interactions (X directly affects Y which affects directly Z => X indirectly affects Z) may show up in the data as association. In my opinion this does not mean that methods cannot find interactions, but rather that interaction concepts have been insufficiently defined and delineated.

These lack of conceptual and mathematical definitions of interactions aside, much of this work appears to be technically well-done (upon code inspection) and reasonably building upon previous work. But not defining which interactions one is talking about, what are state variables and how they combine into observed variables, is in my opinion seriously misleading readers (albeit likely involuntarily). Time series methods infer *direct* interactions while one tries here to infer indirect or blended ones, if I understood correctly. If the methods are used outside their application domain, this should in my opinion be stated as such very clearly in the abstract, intro and discussion so as not to mislead readers, who want to know for the most part whether statistical methods can be used inside their application domain.

I now tackle the main points in more detail

1 - What is an interaction? A key aspect of any interaction or causal inference method evaluation is that the interaction (or causal link) concept has to match in the simulation and statistical model for the interaction/causal inference to work. Ref (30), which the authors build upon, define it as a direct dynamical link between compartments in a dynamical system (i.e. there is a link from i to j if a change in state variable X_i modifies dX_j/dt), and show that Granger causality and CCM can find interactions in this context, even though it is more difficult with environmental confounding, including when corrected for (this last point is rightly and well explained by the authors).

Is the pathogen-pathogen interaction that the authors refer to of the same nature, i.e., a non-zero coefficient linking a time derivative of a pathogen-related quantity to another pathogen "density" in a dynamical system? It may be so if the (possibly reconstructed) derivative of the observed variable for pathogen A is a function of the observed variable for pathogen B. Can the authors manipulate the dynamical system to prove that it is actually the case? If not, then the interaction concepts in the dynamical system and between observed incidences better be given different names. There's no reason to be surprised that Granger and CCM can't find pathogen-pathogen interactions if they have no relationship to the dynamical-system-interactions that Granger analysis and CCM (and transfer entropy) should actually find.

2 - The authors have actually not clarified which time series compartments are observed, i.e. what constitutes incidence data, and how this choice may affect the "interaction" inference. From Fig. 1 and 2 I tend to deduce that they have summed up all infected stages with pathogen A and all infected stages with pathogen B (it was not clear from the code). But how to handle coinfection? Is X_II counted in each pathogen? Or is it removed in each case? How does that choice to include or remove coinfections affects statistical inference?

I want to stress again that there is zero theoretical guarantee that an interaction inference method (like Granger or CCM) designed to infer all links between compartments in the full dynamical system (if it is observed) will actually infer links between sums of compartments, while other compartments stay unobserved. In fact, this recent review [Shojaie, A., & Fox, E. B. (2022). Granger causality: A review and recent advances. Annual Review of Statistics and Its Application, 9(1), 289-319.] mentions that Granger analysis should be applied when there is a "Complete system: All relevant variables are assumed to be observed and included in the analysis—i.e., there are no unmeasured confounders." These challenges may be vaguely alluded to in the present manuscript l. 698-703 but I believe that the authors should be much more precise regarding what variables they observe, how they might combine state variables, and which state variables are unobserved.

3 - Interactions between original pathogen state variables may or may not emerge as correlations between observed variables at new timelags as they blend multiple processes. This makes finding that correlative methods (using GAMs, whose equations might also be presented more precisely, with better indexing) infer "statistical links" between pathogen A and B time series better than interaction inference methods less surprising, I think. I am not saying this is what happens here---but it is possible.

Note that direct interactions cannot always be recovered if only part of the system is observed. This connects to the Granger causality review paper mentioned above, but also ecological theory such as Schaffer linking a "true" ecosystem to a more simplified representation with missing variables https://esajournals.onlinelibrary.wiley.com/doi/10.2307/2937321 Some literature on ecological time series models shows which coefficients can be estimated when only part of the system is observed [e.g. Abbott et al. 2009 https://esajournals.onlinelibrary.wiley.com/doi/full/10.1890/08-0487.1 ]. The statistical literature on latent variable models in statistics probably makes similar points.

Perhaps some algebra can be attempted in the present paper so as to guess which type of relationship could be expected between pathogen incidences, and how the direct interactions in the dynamical system might combine with other parameters in the compound interaction parameters inferred from observed variables, with the various methods considered. One useful starting point may be that the derivative of the sum of infected compartments with pathogen A is the sum of derivatives for individual compartements related to pathogen A, for which expressions are presented in the SI. Another idea would be to approximate the continuous-time dynamical systems (linearized if need be) by a VAR(1) model for the full state variables, leading plausibly to a different time series representation of the two pathogen incidences.

**********

**Have the authors made all data and (if applicable) computational code underlying the findings in their manuscript fully available?**

The PLOS Data policy requires authors to make all data and code underlying the findings described in their manuscript fully available without restriction, with rare exception (please refer to the Data Availability Statement in the manuscript PDF file). The data and code should be provided as part of the manuscript or its supporting information, or deposited to a public repository. For example, in addition to summary statistics, the data points behind means, medians and variance measures should be available. If there are restrictions on publicly sharing data or code —e.g. participant privacy or use of data from a third party—those must be specified.

Reviewer #1: Yes

Reviewer #2: Yes

Reviewer #3: Yes

**********

PLOS authors have the option to publish the peer review history of their article (what does this mean?). If published, this will include your full peer review and any attached files.

If you choose “no”, your identity will remain anonymous but your review may still be made public.

**Do you want your identity to be public for this peer review?**  For information about this choice, including consent withdrawal, please see our Privacy Policy.

Reviewer #1: No

Reviewer #2: No

Reviewer #3: No

**Figure resubmission:**

While revising your submission, we strongly recommend that you use PLOS’s NAAS tool (https://ngplosjournals.pagemajik.ai/artanalysis) to test your figure files. NAAS can convert your figure files to the TIFF file type and meet basic requirements (such as print size, resolution), or provide you with a report on issues that do not meet our requirements and that NAAS cannot fix.

After uploading your figures to PLOS’s NAAS tool - https://ngplosjournals.pagemajik.ai/artanalysis, NAAS will process the files provided and display the results in the "Uploaded Files" section of the page as the processing is complete. If the uploaded figures meet our requirements (or NAAS is able to fix the files to meet our requirements), the figure will be marked as "fixed" above. If NAAS is unable to fix the files, a red "failed" label will appear above. When NAAS has confirmed that the figure files meet our requirements, please download the file via the download option, and include these NAAS processed figure files when submitting your revised manuscript.

**Reproducibility:**

To enhance the reproducibility of your results, we recommend that authors of applicable studies deposit laboratory protocols in protocols.io, where a protocol can be assigned its own identifier (DOI) such that it can be cited independently in the future. Additionally, PLOS ONE offers an option to publish peer-reviewed clinical study protocols. Read more information on sharing protocols at https://plos.org/protocols?utm_medium=editorial-email&utm_source=authorletters&utm_campaign=protocols

# Author comment: Author response to Decision Letter 1

**DOI:** 10.1371/journal.pcbi.1013859.r002

5 May 2026

Attachment Submitted filename: response_to_editor_and_reviewers.pdf

# Peer review: Decision Letter 1

**DOI:** 10.1371/journal.pcbi.1013859.r003

**Authors:** Roger Kouyos, Burcu Tepekule

15 Jun 2026

PCOMPBIOL-D-25-02710R1

The limitations of non-mechanistic methods for characterizing pathogen-pathogen interactions: A simulation study

PLOS Computational Biology

Dear Dr. Kramer,

Thank you for submitting your manuscript to PLOS Computational Biology. After careful consideration, we feel that it has merit but does not fully meet PLOS Computational Biology's publication criteria as it currently stands. Therefore, we invite you to submit a revised version of the manuscript that addresses the points raised during the review process.

Please submit your revised manuscript by **17.07.2026** . If you will need more time than this to complete your revisions, please reply to this message or contact the journal office at ploscompbiol@plos.org. When you're ready to submit your revision, log on to https://www.editorialmanager.com/pcompbiol/ and select the 'Submissions Needing Revision' folder to locate your manuscript file.

Please include the following items when submitting your revised manuscript:

* A letter that responds to each point raised by the editor and reviewer(s). You should upload this letter as a separate file labeled 'Response to Reviewers'. This file does not need to include responses to formatting updates and technical items listed in the 'Journal Requirements' section below.

* A marked-up copy of your manuscript that highlights changes made to the original version. You should upload this as a separate file labeled 'Revised Manuscript with Track Changes'.

* An unmarked version of your revised paper without tracked changes. You should upload this as a separate file labeled 'Manuscript'.

If you would like to make changes to your financial disclosure, competing interests statement, or data availability statement, please make these updates within the submission form at the time of resubmission. Guidelines for resubmitting your figure files are available below the reviewer comments at the end of this letter.

As the corresponding author, your ORCID iD is verified in the submission system and will appear in the published article. PLOS supports the use of ORCID, and we encourage all coauthors to register for an ORCID iD and use it as well. Please encourage your coauthors to verify their ORCID iD within the submission system before final acceptance, as unverified ORCID iDs will not appear in the published article. Only the individual author can complete the verification step; PLOS staff cannot verify ORCID iDs on behalf of authors.

We look forward to receiving your revised manuscript.

Kind regards,

Burcu Tepekule

Guest Editor

PLOS Computational Biology

Roger Kouyos

Section Editor

PLOS Computational Biology

**Additional Editor Comments (if provided):**

Thank you for your revised submission and for addressing the reviewers' comments.

Reviewer 1 is satisfied with the revisions, and we appreciate the effort you have put into improving the manuscript.

However, Reviewer 3 still has substantive concerns that require attention before the manuscript can be accepted. The core issue is that the manuscript lacks sufficient mathematical precision in defining and comparing the interaction concepts at play. In particular, the reviewer asks for a clearer, mathematically grounded definition of what constitutes a biological interaction within the ODE model, and a more explicit treatment of how this relates to the interaction concepts captured by the statistical time series methods. Additionally, the framing of Granger causality should be revisited to better reflect its broader applicability beyond VAR-based representations and its compatibility with latent variable and state-space modelling. Finally, the reviewer also flags several wording issues and asks for greater clarity in the GAM equation. The reviewer has provided a detailed roadmap in their comments on how all of these points could be addressed, and I encourage the authors to engage carefully with their suggestions.

I look forward to receiving your revised manuscript.

**Journal Requirements:**

If the reviewer comments include a recommendation to cite specific previously published works, please review and evaluate these publications to determine whether they are relevant and should be cited. There is no requirement to cite these works unless the editor has indicated otherwise.

1) We ask that a manuscript source file is provided at Revision. Please upload your manuscript file as a .doc, .docx, .rtf or .tex. If you are providing a .tex file, please upload it under the item type u2018LaTeX Source Fileu2019 and leave your .pdf version as the item type u2018Manuscriptu2019.

2) Please provide an Author Summary. This should appear in your manuscript between the Abstract (if applicable) and the Introduction, and should be 150-200 words long. The aim should be to make your findings accessible to a wide audience that includes both scientists and non-scientists. Sample summaries can be found on our website under Submission Guidelines:

https://journals.plos.org/ploscompbiol/s/submission-guidelines#loc-parts-of-a-submission

3) We noticed that you used the phrase 'data not shown' in the manuscript. We do not allow these references, as the PLOS data access policy requires that all data be either published with the manuscript or made available in a publicly accessible database. Please amend the supplementary material to include the referenced data or remove the references.

4) We have noticed that you have uploaded Supporting Information files, but you have not included a list of legends. Please add a full list of legends for your Supporting Information files after the references list.

5) Please send a completed 'Competing Interests' statement, including any COIs declared by your co-authors. If you have no competing interests to declare, please state "The authors have declared that no competing interests exist". Otherwise please declare all competing interests beginning with the statement "I have read the journal's policy and the authors of this manuscript have the following competing interests:"

**Reviewers' comments:**

Reviewer's Responses to Questions

**Comments to the Authors:**

**Please note here if the review is uploaded as an attachment.**

Reviewer #1: The authors have addressed all my concerns. The new analysis based on real-world data is a nice addition to the paper.

Reviewer #3: Review of "The limitations of non-mechanistic methods for characterizing pathogen-pathogen interactions: A simulation study"

This a second review. The authors have made a number of modifications to the manuscript in response to my previous comments, with some improvements. However, I believe that the manuscript still falls a bit short regarding the need for precise vocabulary and mathematical equations, and I encourage the authors to try to be more precise whenever possible. What I expected was a mathematical definition of "interaction" in a pathogen-pathogen sense (using the ODE model), with comparison to the sort of interaction concept used in the methods, including mathematical or computational developments to better compare the notions explored here, with changes to the wording to avoid ambiguities. The authors added caveats and a bit of methodology details but there was no major overhaul, in my opinion. I still do not know which parameters encapsulate the biological interaction between strains (this is not written anywhere as far as I can see, and the reader should not have to dig into the SI to see this). The definitions introduced to clarify the concepts in the introduction stay rather vague. I try to explain below why I still believe this to be problematic and what can be done to be more precise.

Mathematical interaction concepts

l. 67-68 the authors write now to clarify what interaction concepts they are using "In this work, we use the term to refer exclusively to interactions arising due to biological, within-host mechanisms." as opposed to statistical correlations (presumably using Pearson, though this is implicit) which many quantitative biologist now refer to as "statistical associations". There are still a very large number of ways that "interaction" in this sentence can be interpreted. I meant instead something near-mathematical like "when state variable X from the model is increased, state variable Y's derivative dY/dt is decreased" or alternatively, which parameters of the ODE model (which hopefully represents decently well real-life dynamic processes) encapsulate the biological interaction.

The authors introduced in the response letter variables H_A and H_B, which first sum and then integrate between t-1 and t state variables X_IS, X_IE, X_II, X_IT, X_IR before being observed with error. They also write this now in the observation model l. 233. Such presentation is better than the previous version but still unsatisfactory: we have a precise formulation of the state variables that are available for statistical inference (the interaction concept that the time series methods might infer) but no clear idea of what constitute a biological interaction in the mechanistic model, and importantly, what is the link to be expected between the two.

Interesting methodological avenues to better understand and compare the interaction concepts here would be:

- what parameters of the ODE system generate the interaction between strains, i.e. what parameters do we have to set to zero so that the interaction between pathogens disappear?

And, to better understand how the information gets degraded as one moves from the "mechanistic" (compartmental ODE) representation to the statistical time series representation (without latent variables), one could explore:

- what is the relationship between the integrated H_A(t) and the "instantaneous" sum (X_IS + X_IE + X_II + X_IT + X_IR)? (e.g. plot a graph of these two quantities using the ODE system)

- what are the state variables that contribute most to the sum, if any?

Additional wording suggestions

Abstract "simple, purely statistical methods" -> "basic correlative methods" [not sure a "purely statistical" method exists] or perhaps "standard correlations". Same remark for l. 66-67 of the introduction.

Other suggestions

Granger causality is a general concept linked to the increase in variance explained / decrease in residual variance when including a given state variable as covariate in a time series model for another state variable's future values. It could in theory be applied to models including latent variables written in POMP or something else, i.e., there could be other ways to assess Granger causality than with the VAR-based representation used here, although the VAR(p) representation is arguably the most common flavour of such models (and is indeed not appropriate for latent variables). Granger causality as a concept (picking causal state variable based on the fact that they decrease residual variance in state variables to be explained) is very much compatible with mechanistic or latent variable (state-space) modelling. This could be mentioned somewhere. See also your ref 90 Barnett, L., & Seth, A. K. (2015). Granger causality for state-space models. Physical Review E, 91(4), 040101. [This may appear to run counter to the previous statement on confounders by Shojaie & Fox but the latter referred to classical VAR-based Granger Analysis, not latent variable modelling]

The GAM equation l. 386 does not make very clear how time enters the equation. Perhaps making clear the dimensions of the matrices may help.

**********

**Have the authors made all data and (if applicable) computational code underlying the findings in their manuscript fully available?**

The PLOS Data policy requires authors to make all data and code underlying the findings described in their manuscript fully available without restriction, with rare exception (please refer to the Data Availability Statement in the manuscript PDF file). The data and code should be provided as part of the manuscript or its supporting information, or deposited to a public repository. For example, in addition to summary statistics, the data points behind means, medians and variance measures should be available. If there are restrictions on publicly sharing data or code —e.g. participant privacy or use of data from a third party—those must be specified.

Reviewer #1: Yes

Reviewer #3: None

**********

PLOS authors have the option to publish the peer review history of their article (what does this mean?). If published, this will include your full peer review and any attached files.

If you choose “no”, your identity will remain anonymous but your review may still be made public.

**Do you want your identity to be public for this peer review?**  For information about this choice, including consent withdrawal, please see our Privacy Policy.

Reviewer #1: No

Reviewer #3: No

[NOTE: If reviewer comments were submitted as an attachment file, they will be attached to this email and accessible via the submission site. Please log into your account, locate the manuscript record, and check for the action link "View Attachments". If this link does not appear, there are no attachment files.]

**Figure resubmission:**

**Reproducibility:**

To enhance the reproducibility of your results, we recommend that authors of applicable studies deposit laboratory protocols in protocols.io, where a protocol can be assigned its own identifier (DOI) such that it can be cited independently in the future. Additionally, PLOS ONE offers an option to publish peer-reviewed clinical study protocols. Read more information on sharing protocols at https://plos.org/protocols?utm_medium=editorial-email&utm_source=authorletters&utm_campaign=protocols

# Author comment: Author response to Decision Letter 2

**DOI:** 10.1371/journal.pcbi.1013859.r004

14 Jul 2026

Attachment Submitted filename: response_to_reviewers.pdf

# Editor report: Decision Letter 2

**DOI:** 10.1371/journal.pcbi.1013859.r005

**Authors:** Roger Kouyos, Burcu Tepekule

22 Jul 2026

Dear Dr. Kramer,

We are pleased to inform you that your manuscript 'The limitations of non-mechanistic methods for characterizing pathogen-pathogen interactions: A simulation study' has been provisionally accepted for publication in PLOS Computational Biology.

Before your manuscript can be formally accepted you will need to complete some formatting changes, which you will receive in a follow up email. A member of our team will be in touch with a set of requests.

Please note that your manuscript will not be scheduled for publication until you have made the required changes, so a swift response is appreciated.

IMPORTANT: The editorial review process is now complete. PLOS will only permit corrections to spelling, formatting or significant scientific errors from this point onwards. Requests for major changes, or any which affect the scientific understanding of your work, will cause delays to the publication date of your manuscript.

Should you, your institution's press office or the journal office choose to press release your paper, you will automatically be opted out of early publication. We ask that you notify us now if you or your institution is planning to press release the article. All press must be co-ordinated with PLOS.

Thank you again for supporting Open Access publishing; we are looking forward to publishing your work in PLOS Computational Biology.

Best regards,

Burcu Tepekule

Guest Editor

PLOS Computational Biology

Roger Kouyos

Section Editor

PLOS Computational Biology

***********************************************************

# Editor report: Acceptance letter

**DOI:** 10.1371/journal.pcbi.1013859.r006

**Authors:** Roger Kouyos, Burcu Tepekule

PCOMPBIOL-D-25-02710R2

The limitations of non-mechanistic methods for characterizing pathogen-pathogen interactions: A simulation study

Dear Dr Kramer,

I am pleased to inform you that your manuscript has been formally accepted for publication in PLOS Computational Biology. Your manuscript is now with our production department and you will be notified of the publication date in due course.

The corresponding author will soon be receiving a typeset proof for review, to ensure errors have not been introduced during production. Please review the PDF proof of your manuscript carefully, as this is the last chance to correct any errors. Please note that major changes, or those which affect the scientific understanding of the work, will likely cause delays to the publication date of your manuscript.

Soon after your final files are uploaded, unless you have opted out, the early version of your manuscript will be published online. The date of the early version will be your article's publication date. The final article will be published to the same URL, and all versions of the paper will be accessible to readers.

For Research, Software, and Methods articles, you will receive an invoice from PLOS for your publication fee after your manuscript has reached the completed accept phase. If you receive an email requesting payment before acceptance or for any other service, this may be a phishing scheme. Learn how to identify phishing emails and protect your accounts at https://explore.plos.org/phishing.

Thank you again for supporting PLOS Computational Biology and open-access publishing. We are looking forward to publishing your work!

With kind regards,

Sharmila Kamatchi

PLOS Computational Biology | Carlyle House, Carlyle Road, Cambridge CB4 3DN | United Kingdom ploscompbiol@plos.org | Phone +44 (0) 1223-442824 | ploscompbiol.org | @PLOSCompBiol
